# Thyroid-Like Low-Grade Nasopharyngeal Papillary Adenocarcinoma with Biphasic Histology

**DOI:** 10.1155/2020/3275916

**Published:** 2020-01-16

**Authors:** Rusella Mirza, Nestor Dela Cruz, Guillermo A. Herrera

**Affiliations:** ^1^Department of Pathology and Translational Pathology, Louisiana State University Health Science Center, 1501 Kings Hwy, Shreveport, LA 71103, USA; ^2^Department of Pathology, University of South Alabama, 307 N University Blvd, Mobile, AL 36688, USA

## Abstract

Thyroid-like low-grade nasopharyngeal papillary adenocarcinoma (TL-LGNPPA) is a rare primary adenocarcinoma of nasopharynx. The immunohistochemical pattern of this tumor is similar to that of papillary thyroid carcinoma making this neoplasm a challenging diagnosis. A case of TL-LGNPPA with biphasic morphology is presented. The removed tumor from the nasopharynx exhibited a polypoid appearance. Microscopically, it was composed of papillary structures admixed with a few solid areas of spindle cells. The papillae were lined by columnar epithelium. Both the epithelium and the spindle cells were strongly positive for TTF-1 and CK19 and negative for CK5/6, S-100, and thyroglobulin. Cellular atypia, necrosis, and high mitotic rate were absent. Ki67 was less than 2% in the neoplastic cells. No local recurrence or distant metastasis was reported after 12 months of follow-up. Caution should be taken to differentiate TL-LGPPA from other TTF-1-positive neoplasms, especially papillary thyroid carcinoma, as the prognosis for these two tumors is entirely different.

## 1. Introduction

Primary adenocarcinoma of the nasopharynx is extremely rare, representing less than 0.5% of all primary carcinomas of the nasopharynx [1–3]. According to the recent WHO classification, thyroid-like low-grade nasopharyngeal papillary adenocarcinoma (TL-LGNPPA) is considered a variant of nasopharyngeal papillary adenocarcinoma [3]. Patients usually present with nasal fullness, obstruction, or epistaxis. The tumor exhibits exophytic growth with papillary fronds [3].

Immunostains have defined two subsets of low-grade nasopharyngeal papillary adenocarcinoma. The conventional type shows positivity with CK5/6, CK7, and S100. The other subtype is thyroid-like which shows strong positivity for TTF-1 and CK19. Both are CK20 and CDX2 negative.

TL-LGPPA is extremely rare with only about 40 cases reported in English literature. This tumor is composed of papillary structures, and the papillae are lined with columnar or cuboidal epithelium. A bi-phasic pattern with spindle cell population has been reported in very few cases [4, 5].

## 2. Case Report

Our patient is a 54-year-old white male presented with dysphonia since the last three months. He was otherwise healthy. He had been smoking heavily for the last 35 years. No abnormality in thyroid function was detected. Flexible laryngoscopy showed a mass arising from the midline of the anterior nasopharynx. The patient underwent excision of the nasal mass. The mass was tan pink nodular (10 × 5 × 5 mm) and attached with the superior part of the posterior nasal septum. The entire tumor mass was excised by endoscopic surgery. Formalin-fixed, paraffin-embedded tissue samples were cut into 4 *μ*m thick sections and stained with hematoxylin and eosin. Immunostains were performed using the Ventana autostainer (Ventana Medical System Inc., USA) using a panel of antibodies to TTF-1, CK19, CK5/6, S-100, thyroglobulin, and Ki67. Appropriate tissue controls were used for each of the stains.

**Figure 1:**
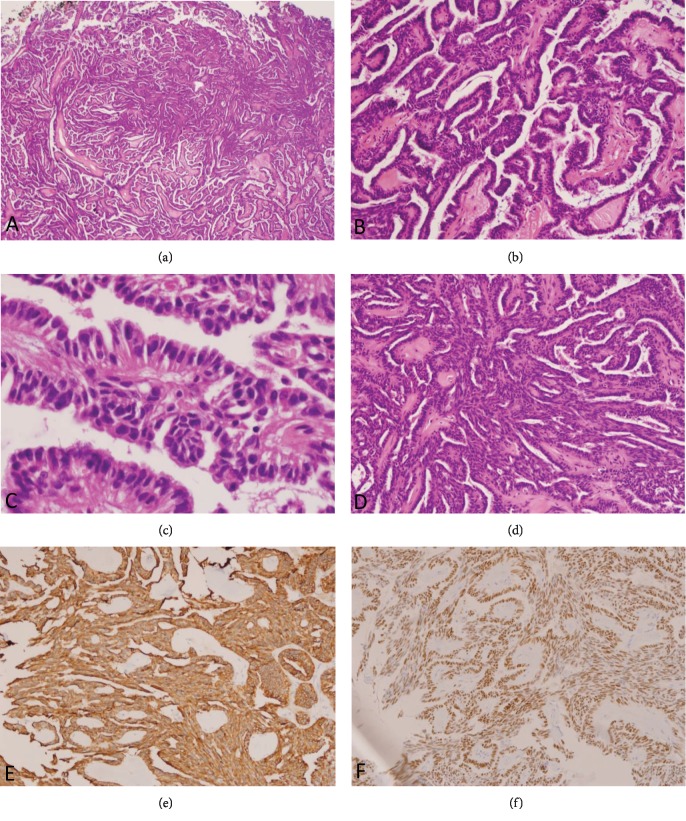
Thyroid-like low-grade papillary adenocarcinoma of nasopharynx demonstrates arborizing papilla (a); papillae have hyalinized fibro vascular core and are lined by a columnar epithelium (b, c). Solid component is made of spindle cells (d). Tumor cells are positive with CK19 (e) and TTF-1 (f).

Microscopically the tumor demonstrated a complex papillary growth with fibrovascular cores, lined by a single layer of columnar to s stratified columnar epithelium (Figures 1(a) and 1(c)). The cytoplasm in the neoplastic cells was scanty and eosinophilic and contained no mucin. The nuclei were elongated and hyperchromatic. No nuclear overlapping, grooving, or pseudonuclear inclusions were observed. Significant hyalinization was present in the fibrovascular cores (Figure 1(b)). The tumor also had several foci with solid aggregates of spindle cells (Figure 1(d)). Immunostains showed strong cytoplasmic positivity with CK19 (Figure 1(e)) and nuclear positivity with TTF-1 (Figure 1(f)) in both epithelial and the spindle cells. Tumor cells were negative for CK5/6, S-100, and thyroglobulin. No significant cellular atypia or mitosis was noted. Ki67 nuclear positivity was seen in less than 2% of the neoplastic cells. The immunomorphologic findings supported the diagnosis of thyroid-like low-grade papillary adenocarcinoma of nasopharynx. No local recurrence has been reported after 12 months of follow-up after excision.

Besides the nasal mass, the reported patient was also found to have a laryngeal mass and a left cheek lesion. The laryngeal mass was subsequently biopsied and diagnosed as an invasive squamous cell carcinoma (T1bN0M0). The patient then received radiation therapy for the laryngeal mass. The cheek lesion was excised and diagnosed as a basal cell carcinoma with negative margins.

## 3. Discussion

Thyroid-like low-grade nasopharyngeal papillary adenocarcinoma (TL-LGNPPA) is a rare variant of primary adenocarcinoma of the nasopharynx. It has an interesting immunostain pattern. Low-grade nasopharyngeal papillary adenocarcinoma was first reported by Wenig et al. in 1988 [6]. Afterwards, Li et al. noticed the TTF-1 expression and reported two cases of thyroid-like low-grade nasopharyngeal papillary adenocarcinoma (TL-LGNPPA) [7]. Since then, few cases have been reported. Literature review demonstrates this tumor typically locates in the nasopharynx arising from the roof or lateral wall of the nasopharynx, and in few cases, it has been noted to be attached with the back end of the nasal septum [6]. In all reported cases, the neoplasms have been exophytic, polypoid masses with or without stalk. The size has ranged from 0.3 cm to 4.0 cm. Because of its location, the patient usually presents with nasal obstruction and/or nasal discomfort, epistaxis, and headache. It has been reported in different age groups, ranging from 9 years to 68 years. Males and females are equally affected. No racial predilection has been reported; however, 26 of 31 cases were reported in Mongolian patients living in China, Taiwan, Japan, and Singapore.

Microscopically, TL-LGPPA is composed of papillary fronds with a fibrovascular core and the papillae are lined by columnar or cuboidal cells. Extensive hyalinization of the fibrovascular core has also been observed by others [3, 4]. Besides the epithelial component, there may be a second cell population of spindle cells. There are few other reports of this kind of biphasic pattern, and the presence of spindle cells does not have any prognostic value [4]. TL-LGPPA is a great mimicker of PTC. We can find rare PTC-type nuclear features in TL-LGPPA [4, 5]. The neoplastic cells are positive with TTF-1, CK19, HBME-1, EMA, and vimentin [8]. However, cells are negative with thyroglobulin and Pax8 immunostain which is helpful to differentiate it from PTC. In our case, multiple foci with solid aggregates of spindle cells were noted and both the epithelial and spindle cells were strongly positive for TTF-1 and CK19. There is no reported association of TL-LGPPA with Epstein-Barr virus (EBV) or human papilloma virus (HPV) [9, 10]. Molecular studies have been conducted in few cases and did not show any genetic alteration in BRAF, RAS, EGFR, and ALK gene [3, 5].

TL-LGPPA is a low-grade malignancy. No lymph node metastasis or distant metastasis has been reported. Radical excision of the tumor is the major treatment modality. Due to the few case reports in the literature, it is difficult to make definitive conclusions. However, the follow-up of all reported cases demonstrates no evidence of local recurrence or distant metastasis [3, 4].

## 4. Conclusion

TL-LGPAA is a rare variant of papillary adenocarcinoma of the nasopharynx. The morphological and immunohistochemical pattern is almost similar to that of papillary thyroid carcinoma. The inclusion of thyroglobulin and Pax8 immunostains is recommended together with TTF-1 and CK19. Caution should be taken as the treatment and prognosis are entirely different in these two neoplasms.
